# The ethical landscape of robot-assisted surgery: a systematic review

**DOI:** 10.1007/s11701-025-02228-1

**Published:** 2025-03-06

**Authors:** Joschka Haltaufderheide, Stefanie Pfisterer-Heise, Dawid Pieper, Robert Ranisch

**Affiliations:** 1 Juniorprofessorship for Medical Ethics with a focus on Digitization, Faculty for Health Sciences Brandenburg, University of Potsdam, Am Mühlenberg 9, Potsdam, 14476 Brandenburg Germany; 2 Institute for Health Services and Health System Research, Center for Health Services Research Brandenburg, Faculty of Health Sciences Brandenburg, Brandenburg Medical School Theodor Fontane (MHB), Potsdam, Germany

**Keywords:** Robot-assisted surgery, Ethics, Ethical issues, Systematic review, Surgical ethos, Health technology

## Abstract

Robot-assisted surgery has been widely adopted in recent years. However, compared to other health technologies operating in close proximity to patients in a vulnerable state, ethical issues of robot-assisted surgery have received less attention. Against the background of increasing automation that is expected to raise new ethical issues, this systematic review aims to map the state of the ethical debate in this field. A protocol was registered in the international prospective register of systematic reviews (PROSPERO CRD42023397951). Medline via PubMed, EMBASE, CINHAL, Philosophers’ Index, IEEE Xplorer, Web of Science (Core Collection), Scopus and Google Scholar were searched in January 2023. Screening, extraction, and analysis were conducted independently by two authors. A qualitative narrative synthesis was performed. Out of 1723 records, 66 records were included in the final dataset. Seven major strands of the ethical debate emerged during the analysis. These include questions of harms and benefits, responsibility and control, professional-patient relationship, ethical issues in surgical training and learning, justice, translational questions, and economic considerations. The identified themes testify to a broad range of different ethical issues requiring careful deliberation and integration into the surgical ethos. Looking forward, we argue that a different perspective in addressing robotic surgical devices might be helpful to consider upcoming challenges of automation.

## Background

Robot-assisted surgery (RAS) has been widely adopted in recent years [1–4]. RAS involves the use of sophisticated robotic platforms during invasive surgical procedures to support or assist surgeons, enabling high accuracy and precision [5]. Currently, around 40 different robotic systems are commercially available [6] and are used in various surgical subfields. The most commonly employed is the Da Vinci surgical system, with roughly 6,000 devices in operation worldwide that have, up to now, performed approximately 8.5 million procedures [7].

Although the origins of RAS can be traced back to the 1970s, with commercially available devices emerging in the 1990s [8, 9], it is particularly in the last decade that case volumes have increased significantly, and RAS has diffused into general surgical practice [2, 10, 11]. Especially in the USA, UK, Germany and Japan, growing case volumes have been observed while RAS has become the standard of care in various procedures [7].

Compared to other health technologies that operate in close proximity to vulnerable care recipients, the development and introduction of RAS has received relatively little attention from both ethicists and the public. Ethical issues can be broadly understood as situations that require a value judgement. Consequently, ethical issues cannot be settled based on factual knowledge alone but require claims about what is good and right to guide further action. These situations may include questions such as how to protect patients’ wellbeing and autonomy, questions of surgeons’ responsibilities towards patients undergoing RAS or questions regarding the professional ethos and role of robotic surgeons in light of new technological innovation. Given its long history and its prevalence in surgical practice, it is surprising that RAS has not been accompanied by a broader debate. Ethical investigations have been dispersed across various academic fields, including philosophy, medical ethics, philosophy of technology, medicine, and computer sciences. Recent technological advances have, however, sparked a renewed interest in these issues. Commentators have highlighted their understanding of RAS as a potential bridge technology, linking traditional laparoscopic approaches with more advanced technical arrangements in surgery. This includes, for example, using available data from the machines to evaluate and optimize surgical workflows, combining RAS image data and artificial intelligence (AI) to further the development of tissue recognition and computer-based surgical automation [12] as well as enabling more complex forms of interaction between surgeons and machines [13].

Increasing automation, the use of more advanced devices, and evolving patterns of interaction will inevitably raise a variety of new ethical and social questions. This review is the first step in an ongoing project that aims to anticipate and evaluate potential ethical issues of cutting-edge and next-generation devices. It compiles the current state of knowledge and understanding of ethical issues associated with RAS, drawn from both experience and scholarly discussions on current technology. The goal is to establish a comprehensive foundation that maps ethical perspectives and value considerations, which can then inform discussions surrounding potential future developments. To this end, we conducted a comprehensive systematic review addressing the following research question:

1. What are the ethical issues surrounding the existing use of robot-assisted surgery?

This includes investigating the main themes of the ethical debate and mapping how technical properties and perspectives on these devices are connected to these themes. With the latter, we aim to understand how specific features of RAS are problematized from an ethical perspective to allow for a more in-depth understanding of the ethical debate.

First, we provide an overview of our methods for conducting this review. We then present the results of our analysis, focusing on the main themes that can be identified in the current literature. In discussing the implications of these findings, we highlight their tight interconnectedness and suggest to examine these questions through the lense of the surgical ethos. Finally, we comment on two different frames as to how RAS is addressed in ethical debates. We suggest that understanding and reflecting on these frames might be especially helpful with a view to future debates.

## Methods

Systematic reviews of ethical issues differ from established approaches in empirical sciences which cannot be transferred to ethical questions directly [14]. Droste et al. have argued that this requires to organize key concepts for the search differently while resorting to accepted principles of information retrieval. In following Droste et. al and their arguments [15], we, hence used the adapted PIE (Patient/Problem, Intervention, Ethical Issue) scheme instead of the commonly used PICO scheme (Patient/Problem, Intervention, Comparison, Outcome) to determine our key concepts. In reporting, we follow the guideline for reporting systematic reviews in ethics (RESERVE) [14]. A review protocol was designed and agreed upon by the authors. It was registered in the international prospective register of systematic reviews (PROSPERO) [16]. The goal was to analyze ethical issues emerging from practical experience, such as those documented in empirical work, case reports or letters, as well as from theoretical work from different scholarly perspectives.

### Inclusion and exclusion criteria

We did not define inclusion or exclusion criteria based on patient population or scope of the problem since we wanted to gather all available information on RAS. Instead, we used two key concepts based on the nature of the intervention and the nature of ethical issues to define our inclusion criteria. The first, RAS was defined as a surgical procedure with any surgical technology that places a computer-aided electromechanical device in the interactional path between surgeon and patient and "assumes some degree of control heretofore reserved for the surgeon." [17]. We distinguished computer-aided devices from purely mechanical manipulators based on their capacity for complex digital data processing. A robotic surgical device, therefore, is any electrical system capable of information processing that incorporates programmable actuator mechanisms to facilitate in the placement of surgical instruments within a patient. This definition includes all settings in which surgeons make use of any kind of computer-aided manipulator (actuator) to enhance their abilities while the traditional local direct contact between surgeons and patients is altered. The definition excludes purely mechanical manipulators as well as advanced information processing systems without physical components for manipulation such as artificial intelligence navigation systems for laparoscopic surgery

We excluded all papers discussing exclusively hypothetical technical arrangements such as fully autonomous RAS to avoid discussions not grounded in experience and technical realities. Operationalizing this criterion proved more difficult than expected. We planned to use a taxonomy for levels of functional autonomy, following the categorization of Yang et al. [18], subsequently refined by Lee et al. [19]. However, drawing on this approach turned out to be difficult, as many authors in our records made only loose reference to specific technical features, merely implied a certain state of technology or discussed various scenarios. Consequently, we resorted to an array of criteria as proxy indicators. We checked whether and which devices were referenced by name, or whether reference to real world cases was made. We hypothesized that this would keep us in the range of commercially available and approved devices. Where no device could be identified, the state of development (e.g. prototypes, technical concept studies) or the reference to technical features was unclear, we checked and discussed whether described or implied features would lie within the autonomy levels 0 and 2 according to Yang et al. In case of reference to various states of technology (e.g. future and present) the work was included but only relevant parts were extracted.

For our second key concept, ethical issues, we defined these as a state in which the moral implications of a given situation cannot be determined without much reservation, where there is disagreement regarding the right course of action, or conflicting moral obligations or values are present [20, 21]. Broadly speaking, an ethical issue requires value judgements about what is right or wrong and cannot be resolved by reference to facts or experience alone. Important values and principles to consider may include the patients’ autonomy and wellbeing (raising, for example, questions of informed consent, privacy and confidentiality), patients’ right to equitable treatment (raising questions about access to and distribution of RAS) or duties and responsibilities of surgeons based on their professional ethos. Ethical issues can include a broad range of situations in different states, such as unclear or undetermined benefits, chances, risks, or harms; unclear, undetermined or conflicting views about addressees of moral complaints or bearers of moral significance as well as situations in which moral principles were evidently overlooked or ignored. We determined an ethical issue to be present whenever it was recognized as such in the literature or was identified during analysis based on our criteria.

We did not define any additional inclusion or exclusion criteria based on, for example, specific patient groups, type of publication or type of work presented.

### Sources

Database searches were conducted in January 2023. Databases included Medline via PubMed, EMBASE, CINHAL, Philosopers’ Index, IEEE Xplorer, Web of Science (Core Collection), Scopus, and Google Scholar. In Google Scholar, we limited the search to the first 200 records. In addition, we searched citations of included full-text articles, scanned conference proceedings, and consulted with experts from the field. Details on the search strategy can be found in the protocol [22]. An overview and example search string is given in Table 1.

**Table 1:** Overview on the sources and searchstring

Sources
Medline via PubMed
EMBASE
CINHAL
Philosophers’ Index
IEEE Xplorer
Web of Science (Core Collection)
Scopus
Google Scholar

Example search string (PubMed)
1. Robotic surgical procedures[MeSH Terms]
2. Surgical[tiab]
3. Surgery[tiab]
4. Minimally invasive[tiab]
5. Laparoscopy[tiab]
6. Micro[tiab]
7. 1. OR 2. OR 3. OR 4. OR 5. OR 6.
8. Robot*[tiab]
9. Robotic[tiab]
10. Robot assisted[tiab]
11. Robot-assisted[tiab]
12. 8. OR 9. OR 10. OR 11.
13. Ethics[MeSH Terms]
14. Ethic*[tiab]
15. Ethical*[tiab]
16. Moral*[tiab]
17. 13. OR 14. OR 15. OR 16.
18. 7. AND 12. AND 17.

### Screening

Two of the authors [JH and SPH] independently screened titles and abstracts. Conflicts were resolved through discussion. In case of prevailing disagreement in the title and abstract stage, the decision was postponed until the full-text screening stage. Full texts were screened independently by the same authors. Disagreement was resolved through discussion.

### Extraction and analysis

Data was subsequently extracted by the same authors using a self-designed extraction form. Besides basic bibliographic information, the authors extracted data on interventions and settings, information about devices referenced, ethical values named or referenced, ethical arguments, key conclusions, and further recommendations. For data extraction, these categories were translated into a preliminary coding tree using MAXQDA 20.

### Synthesis

A synthesized coding tree using the above-named categories was developed, based on the first ten records in alphabetical order to ensure a shared understanding of the coded features. These first ten records were screened independently by two authors, and the results and coding schemes were jointly discussed. Following the quality appraisal procedure as outlined below, the first author coded all remaining papers and at the same time categorized them to steer further extraction. The second author, then independently coded all records of higher quality and reviewed the rest for saturation with the help of a supervised assistant. Finally, all codes and categories were synthesized.

### Quality appraisal

Currently, no agreed upon criteria for the quality appraisal of normative literature exist. However, we agree with Mertz et al. in that quality appraisal is an important step in systematically reviewing literature [23]. Based on these considerations, we developed a hybrid strategy.

**Table 2:** Criteria for hybrid quality assessment

**1. Weighing quality in extraction**			
Is the publication peer-reviewed or otherwise quality controlled during the publication process?			
Is the publication a comment, perspective, letter, viewpoint or editorial?			
Does the content of the publication display material richness?			
**2. Weighing Quality in Synthesis**			
Does the argument display a focused question?			
Does the argument relate/engage with existing literature?			
Does the argument denote a normative framework/principle?			
Does the argument refer to empirical data in its descriptive claims?			
Does the argument reflect on its assumed relationship of normative and empirical claims?			
Does the argument come to a conclusion?			
**3. Weighing Quality in Reporting**			
Does the dataset/further literature display arguments or objections contrary to its position?			
Does the dataset/literature indicate limits in generalizability or absoluteness of a claim?			
Does the dataset/further literature display empirical evidence contrary to the displayed empirical claims?			

Given the aim to steer the process of reviewing towards a higher level evidence as well as the fact that content-related methods of appraisal require to be included in the later stages of the process, we integrated quality criteria in the extraction and analysis as well as the synthesis and reporting stage giving increasing weight to content-related criteria in the later steps [23]. A first step of our quality appraisal served to steer the extraction and analysis stage of our review and was based on procedural criteria. We used this step to inform the grouping of records in those which would be primarily extracted and those which were used to check for saturation, hence giving weight to those included studies that indicate to be epistemically more trustworthy and display a certain material richness in content. In the second step, we used a modified version of the reporting criteria as proposed by McCullough to appraise comprehensiveness and validity of the extracted information pieces [24]. This step was used to inform the synthesis and determine to what extent the synthesis should rely on an extract. Given the interdisciplinary nature of medical ethics research, we took into consideration that arguments in this field often do not only rely on purely normative considerations but include so-called mixed judgements which encompass factual or empirical claims as well as normative statements to arrive at a conclusion [25]. We hence modified the approach of McCullough to reflect this based on current proposals of reporting empirically informed ethical judgements. For the final step we followed McDougall who suggests to understand quality appraisal as integral part of the reporting of normative systematic reviews [26]. We hypothesized that this stage should rely on content-based criteria of critical thinking and to report strength and limitations of claims made in our dataset as well as to contextualize findings with the wider literature if available. Table 2 shows the leading questions of our assessment in each stage. The first two steps were carried out by the first author, [JH], and were supervised by [RR and DP]. The third step was carried out in a joint review session with all authors.

## Results

### Description of included studies

The initial search resulted in 1,723 records with 955 remaining after removal of duplicates; 795 records were excluded during title and abstract screening.

**Fig. 1:**
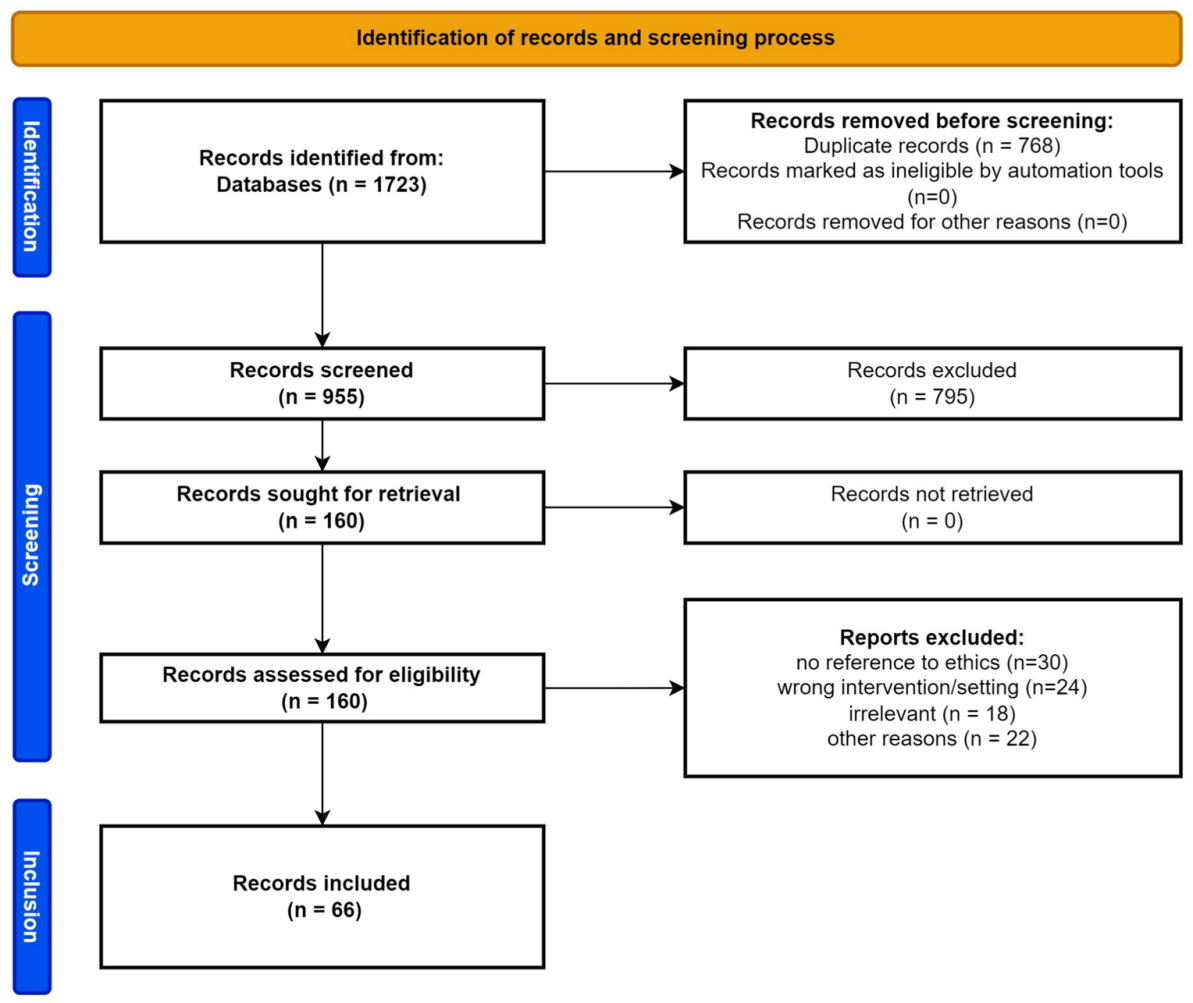
Flow of studies through the screening process

For 160 records, the full text was accessed. 94 were excluded, mostly because of missing reference to ethical issues and wrong settings or interventions. 66 records were included in the final dataset. The flow of records through the screening process can be seen in Fig. 1. Our dataset included 27 original articles, six book chapters, 11 reviews, eight comments, editorials or letters, eight perspective articles and six articles of unclear type. 45 publications were published in the field of medicine including 24 in surgery. Ten publications came from the field of ethics, applied ethics and neighbouring fields. Eight works were published in the field of the technical sciences. Two studies came from social and behavioural sciences. One work was of other origin. Publication dates ranged from 2000 to 2022. Table 3 gives an overview. Table 4 shows the procedural quality criteria as outlined above.

Of the 66 included publications, 21 were classified as being of higher quality, that is, being peer-reviewed and displaying a certain material richness, and were therefore assigned a greater weight during extraction and synthesis (compared to 45 not being peer-reviewed, not displaying material richness, or both).

**Fig. 2:**
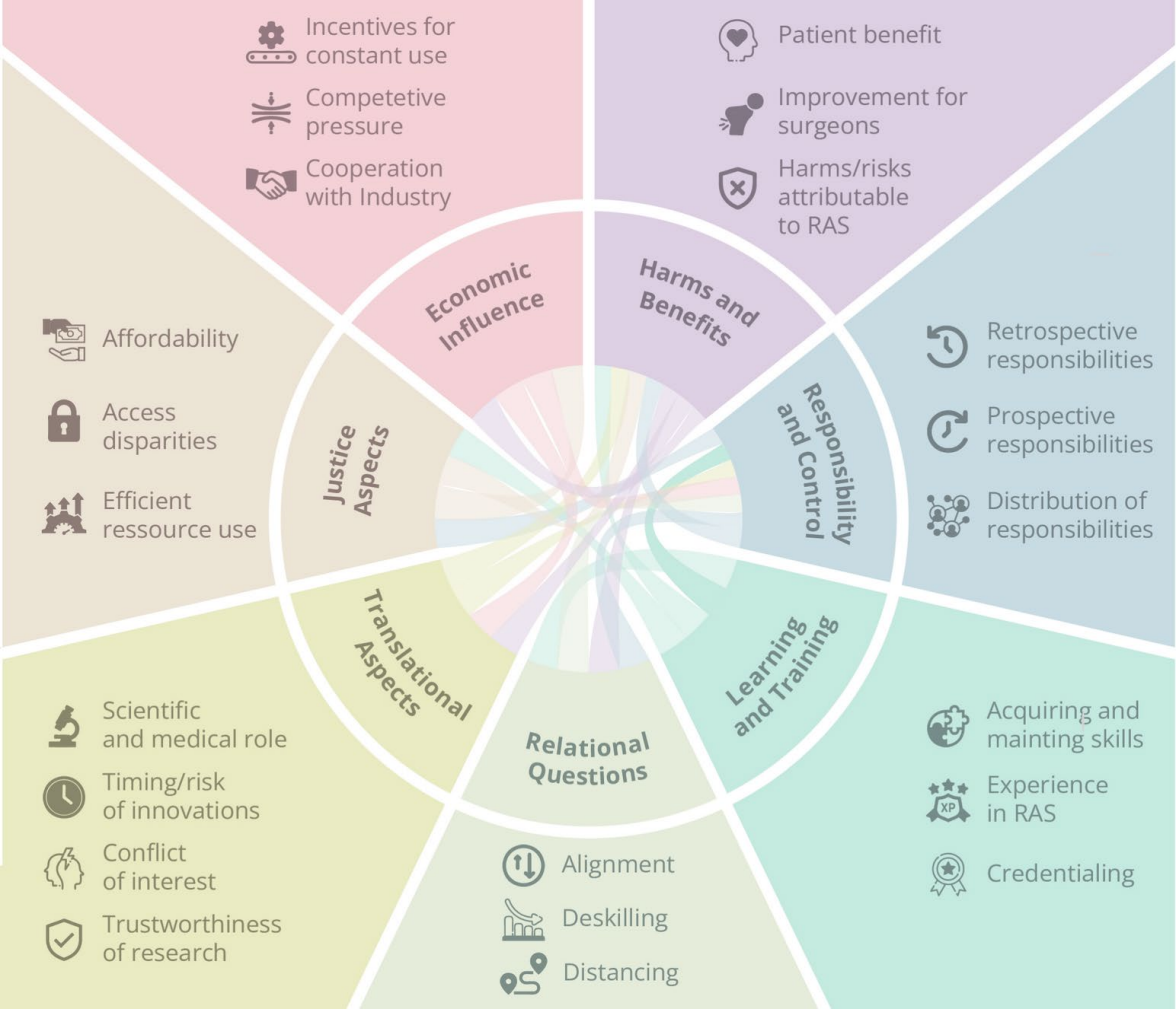
The landscape of ethical issues in RAS

With our analysis, 7 major strands of the debate emerged. These include questions of harms and benefits, responsibility and control, the professional-patient-relationship, ethical questions of surgical training and learning, questions of justice, translational questions and questions regarding economic values. In what follows, we base our report on these major strands noting a tight interconnectedness of all themes that is illustrated in Fig. 2. We use these connections to further structure our report and provide a consistent (though surely not the only possible) narrative.

### Mapping the ethical landscape

#### Benefits and harms to patients and surgeons

With regard to patient benefit, it is agreed that it is an important ethical criterion in support of the use of RAS [27, 28]. Wightman et al. argue that patient benefit should be the primary evaluation criterion from an ethical perspective [29]. The assumed benefits include, for example, lower rates of postoperative complications [30], a shortened length of stay in hospital, a reduction of care expenses [31], less invasive procedures [30, 32] as well as an overall increase in the quality of surgical procedures through standardization and prevention of outliers in performance [33, 34]. In addition, these devices may enable more complex procedures that would be difficult or impossible to handle with traditional methods [29, 35, 36] and may increase the precision of surgical maneuvers [37]. In some cases, further benefits may extend beyond the procedure itself, such as a decreased exposure to radiation or a reduced risk of infections [30, 33]. However, Decker cautions that evaluations of these benefits must be highly specific to each surgical domain and should not be generalized [27]. This caution is echoed by several authors noting an unclear situation regarding the question whether these assumed benefits can actually be supported by evidence in specific cases [38–40]. Hutchinson et al. note a considerable debate with regard to these issues, concluding that establishing the superiority of robot-assisted procedures beyond doubt may be a challenge [41]. According to Scheetz and Dimick, commenting on a safety communication released by the United States Food and Drug Administration (FDA) [42], there is little robust evidence suggesting that robot-assisted surgery is superior to laparoscopic or open procedure [43, 44]. While the FDA communication is mainly concerned with mastectomy and other cancer-related surgery, the authors cite further evidence to generalize this claim.

**Table 3:** Overview on the included records

Title	Focus of study	Journal category*	Article type**	Year	COI
Angelos [45]	Surgical ethics, RAS procedure	N.a. (Endocrinology, surgery)	P/V	2015	No
Angelos [32]	Ethics	Surgery	P/V	2016	No
Angelos [46]	Ethics	Surgery	E	2017	Unclear
Anvari [47]	Telesurgery, surgical robots	Respiratory System; Surgery; Cardiac and Cardiovascular System	R	2005	Unclear
Bendel [48]	Ethics, Medical Machine Ethics	N.a. (Medical Ethics)	Ch	2015	Unclear
Cavinatto et al. [34]	Conflict of Interest, RAS Procedure, Medical Ethics	Orthopedics	OA(e)	2019	Yes
Chitwood [49]	Ethics	Surgery; Cardiac and Cardiovascular System; Respiratory System	Co	2019	No
Collins et al. [50]	Telesurgery, Education, Patient Safety	Urology and Nephrology	R	2020	Yes
Criss et al. [51]	Conflict of Interest, Funding	Surgery	R	2019	Unclear
Datteri [28]	Medical Robotics, Robot Ethics, Philosophy of Science	Ethics	OA(t)	2013	Unclear
De Togni et al. [52]	Artificial Intelligence, Health and Care, Robot Ethics	Public, Enivironmental and Occupational Health	OA(t)	2021	Unclear
Decker [27]	Robotics, Ethics, Hospital	N.a. (Medical Ethics)	OA(t)	2018	Unclear
DeFrance et al. [53]	RAS Procedure, Outcomes	Orthopedics	R	2021	Yes
Di Paolo et al. [54]	RAS, inequality, Informed Consent	Surgery	O	2019	No
Dickens & Cook [55]	RAS, Legal issues, Ethics across borders	Obstetrics and Gynecology	OA(t)	2006	Unclear
El-Bahnasawi et al. [56]	Innovation, Training	Surgery; Gastroenterology and Hepatology	L	2019	No
Esperto et al. [31]	RAS in Urology, Ethical challenges	Urology and Nephrology	R	2022	No
Ficuciello et al. [57]	RAS, Ethics, Human Control	N.a. (Behavioural Robotics)	OA(t)	2019	Unclear
Geiger & Hirschl [58]	Innovation, RAS, Ethics	Surgery; Pediatrics	OA(t)	2015	Unclear
Giovagnoli et al. [35]	RAS, Robotics, Roboethics	Philosophy	OA(t)	2019	Unclear
Graur et al. [59]	Ethics, RAS, telesurgery	N.a. (Computer Science, Hardware)	Ch	2010	Unclear
Hung et al. [60]	RAS, telemedicine, education	Urology and Nephrology	R	2018	No
Hutchison et al. [41]	Justice, Innovation, Ethics	Medical Ethics	OA(t)	2016	Unclear
Jimbo et al. [61]	Conflict of Interest, RAS in Urology, Robotics	Urology and Nephrology	OA(e)	2019	Yes
Jones & McCullough [62]	Ethics	Surgery; Peripheral Vascular Diseases	O	2002	Unclear
Lam et al. [63]	Digital Surgery, Robotics, Ethics	Health Care Sciences and Services; Medical Informatics	P/V	2021	Yes
Larson et al. [64]	Ethics, Patient Safety	Surgery	OA(t)	2014	Yes
Lee Char et al. [65]	Informed Consent, Innovation	Surgery	OA(e)	2013	No
Lin et al. [66]	Robotics	N.a. (Technology, engineering, machinery)	Ch	2012	Unclear
Lirici [44]	RAS, Innovation	Surgery	E	2022	No
Mavroforou et al. [67]	Robotics, Surgery, Ethics	Peripheral Vascular Diseases	O	2010	Unclear
Narsinh et al. [33]	Robotics, Ethics, Telemedicine	Neuroimaging	OA(t)	2022	Yes
Nestor & Wilson [36]	Robotics, Surgery, Anticipatory Ethics	Ethics	OA(t)	2019	Unclear
Patel et al. [68]	RAS, Spin	Gastroenterology and Hepatology; Surgery	OA(e)	2015	Yes
Patel et al. [69]	RAS, Conflict of Interest	Surgery	OA(e)	2018	Yes
Platis, Zoulias [30]	Healthcare Services, RAS, Administration	N.a. (Social and behavioural sciences)	OA(t)	2014	Unclear
Polk et al. [70]	Conflict of Interest, Guidelines	Gastroenterology and Hepatology; Surgery	P/V	2019	Yes
Saniotis & Henneberg [37]	Neurosurgical Robot systems, Moral Agency, Bioethics	N.a. (Ethics)	OA(t)	2021	No
Satava [71]	Legal Issues, Ethical Issues	N.a. (Surgery)	OA(t)	2002	Unclear
Schlottmann & Patti [72]	Simulation, Training, RAS	Surgery	L	2017	No
See et al. [73]	RAS, Commercial Endorsement, Case Volumes	Oncology; Urology and Nephrology	OA(t)	2014	Unclear
Senapati & Advincula [74]	Robotics, Telesurgery, Gynecologic Surgery	Obstetrics and Gynecology	P/V	2005	Unclear
Shahzad et al. [75]	Robotics, Tele-Robotics, Ethical Issues	Medicine, General and Internal	R	2019	No
Sharkey & Sharkey [38]	RAS, Ethics	N.a. (Ethics)	Ch	2012	No
Sharkey & Sharkey [76]	RAS, Ethical framework	Computer Science, Hardware and Architecture; Computer Science, Software Engineering	OA(t)	2013	No
Sheetz & Dimick [43]	RAS, Patient Safety, Learning	Medicine, General and Internal	P/V	2019	Unclear
Siciliano & Tamburrini [77]	Robotics, Ethics, Human–Machine-Interaction	Religion	OA(t)	2019	Unclear
Siqueira-Batista et al. [78]	Bioethics, Surgery, Robotics	Gastroenterology and Hepatology	R	2016	No
Smith [79]	RAS, Responsibility	Urology and Nephrology	E	2013	Unclear
Smyth et al. [40]	RAS, Surgical Ethos	Surgery; Respiratory System; Cardiac and Cardiovascular System	P/V	2013	No
Spiers et al. [80]	RAS Kidney transplant, minimally invasive surgery	Urology and Nephrology	R	2022	Yes
Spillman & Sade [81]	Ethics	N.a. (Medical Ethics)	O	2014	Unclear
Stanberry [82]	Telesurgery and Tobotics, health telematics	Medicine, General and Internal	OA(t)	2000	Unclear
Steil et al. [83]	Robots, team-machine-interaction, hybrid action	Health Care Sciences and Services; Medical Informatics; Computer Science, Information Systems	O	2019	No
Strong et al. [84]	Surgical Education, Human/Robotic, Costs	Surgery	O	2014	Yes
Sullins [85]	Ethics, Trust, RAS	N.a. (Philosophy)	OA(t)	2014	Unclear
Swarnalatha & Menon [86]	Telerobotics, medical robotics	N.a. (Engineering, information technology)	OA(t)	2022	Unclear
Teller et al. [39]	RAS, Innovation, Ethics	Surgery; Oncology	OA(t)	2022	No
Thomas et al. [87]	Robotics, Advertising, Surgery	Surgery	R	2020	Yes
Tzafestas [88]	Robotics	N.a. (Engineering, information technology)	Ch	2016	Unclear
van der Waa et al. [89]	Moral Decision Making, Human-Agent Teaming, Machine Ethics	N.a. (Engineering, behavioural sciences)	Ch	2020	Unclear
Vilanilam & Venkat [90]	Robotic Neurosurger, Ethics, Legal Issues	Surgery; Clinical Neurology	E	2022	No
Whiteside [91]	Robotic Gynecologic Surgery	Obstetrics and Gynecology	E	2008	Yes
Wightman et al. [91]	Ethics, RAS, informed consent	Surgery	R	2020	Yes
Woo et al. [92]	Informed Consent, Surgeon Experience	Surgery; Cardiac and Cardiovascular System; Respiratory System	P/V	2019	No
Zorn et al. [93]	Urologic Robotic Surgery, Training, Credentialing	Urology and Nephrology	OA(t)	2009	Yes

$^{*}$ By Web of Science Journal Classification (n.a. is self-assessed)

$$^{**}$$ P/V = Perspective/Viewpoint, E = Editorial, R = Review, Ch = Chapter, OA(e) = Original Article (empricial), OA(t) = Original Article (theoretical), L = Letter, C = Comment, O = Other

Authors disagree on the reasons for the perceived lack of empirical evidence supporting arguments based on patient benefit. In many cases, determining specific outcomes (e.g. beyond decreased morbidity and mortality) is challenging [45]. Whether, for example, advantages such as improved accuracy of maneuvers correlate with better patient outcomes is uncertain [53]. Furthermore, a lack of scientific quality of existing studies is noted. According to Scheetz and Dimick, for example, most studies in the field have only been small, single-center trials without effective controls [34, 43], while long term data is often unavailable. Consequently, there is little evidence with regard to patient benefits [43, 44, 90]. Another noted factor concerns the trustworthiness of research on RAS [88], which may be influenced by the close intertwinement of researchers and industry. Failure to disclose financial connections and conflict of interest is also common and contributes to a lack of transparency [51, 61, 68, 70, 94]. Several authors report correlations between ties to the industry and more favourable study outcomes or a tendency to overinterpret results and exaggerate claims [51, 68, 94]. Although the validity and interpretation of these findings is itself subject to debate [95, 96], it shows ongoing concerns with regard to transparency and trustworthiness of empirical results.

The potential harm for patients is primarily discussed in terms of risks to physical integrity [28, 29, 31, 38, 43, 58, 66, 90]. Although potential harm is frequently cited, the exact nature of these risks remains unclear and is only roughly indicated. Wightman et al. argue that devices should not add additional harm (or the risk of it) to a procedure [29]. Discussing a specific procedure, Angelos delivers an example, noting that robotic procedures can be connected to very specific risks that come with the positioning of patients and the point of access of the instruments that is unique to RAS. As Angelos concludes, this results in specific risks that are directly attributable to the use of the device [45]. Sharkey notes increased morbidity and mortality as well as longer hospital stays but does not provide backing evidence for these claims [76]. Sheetz and Dimick cite evidence for radical hysterectomy showing a significant decrease in long-term survival rates [43]. Others note that harm due to malfunctions can only be observed in a minority of cases [28, 31]. Di Paolo et al. nevertheless caution that 77% of adverse events in RAS with DaVinci Systems could be rated as device-specific incidents, that is, situations in which the device played a significant and causally relevant role [54]. This claim is based on data from 2009 to 2012, including all adverse events reported in the FDA MAUDE Database. However, as this data also includes incidents occurring in preparation for surgery, we find that the cited evidence does not yield direct insights towards answering the question as to how many incidents in which harm was very likely can be attributed to the devices. We conclude that it does not support Di Paolo et al’s claim. Notwithstanding this, the data shows that at least around 25% of all incidents have moderate (17,73%), severe (4,44%) or life-threatening outcomes (2,89%) implying patient involvement. It is, hence, still likely that in a considerable amount of negative outcomes a causal connection to the use of RAS exists [97]. In addition, Geiger and Hirschl suggest, based on empirical evidence, that complications and malfunctions are frequently underreported [58].

Beyond physical risks, informational harms are also widely discussed in the literature. These include threats to privacy, confidentiality or security breaches that are primarily connected to data collection and use that comes with the devices [75, 76, 81]. Data security is mostly discussed within the context of telesurgery, a subspeciality of RAS, in which the distance between the control console and the actuator extends the usual distances and can even expand beyond national borders [38, 47, 76]. It should be noted, however, that the underlying cause for security vulnerabilities is the transmission of data between device and control. Remote distance may therefore add only additional layers of vulnerability and complexity to an already existing problem [98]. In this regard, Vilaniam and Venkat argue that clinical data security concerns arise whenever third parties are involved that could access, exploit or misuse data [90].

**Table 4:** Overview on procedural quality control criteria

Paper	Peer reviewed	Minor work$^*$	Material richness
Angelos [45]	Yes	No	Yes
Angelos [32]	Yes	No	Yes
Angelos [46]	No	Yes	Yes
Anvari [47]	Yes	No	Yes
Bendel [48]	Unclear	No	No
Cavinatto et al. [34]	Yes	No	Yes
Chitwood [49]	No	Yes	No
Collins et al. [50]	Yes	No	No
Criss et al. [51]	Yes	No	Yes
Datteri [28]	Yes	No	Yes
De Togni et al. [52]	Yes	No	Yes
Decker [27]	Yes	No	Yes
DeFrance et al. [53]	Yes	No	Yes
Di Paolo et al. [54]	No	Yes	Yes
Dickens & Cook [55]	Yes	No	No
El-Bahnasawi et al. [56]	No	Yes	No
Esperto et al. [31]	Yes	No	Yes
Ficuciello et al. [57]	Unclear	No	Yes
Geiger, Hirschl [58]	Yes	No	Yes
Giovagnoli et al. [35]	Unclear	No	No
Graur et al. [59]	Unclear	No	Yes
Hung et al. [60]	Yes	No	No
Hutchison et al. [41]	Yes	No	Yes
Jimbo et al. [61]	Yes	No	No
Jones & McCullough [62]	No	Yes	No
Lam et al. [63]	No	Yes	No
Larson et al. [64]	Yes	No	No
Lee Char et al. [65]	Yes	No	Yes
Lin et al. [66]	Unclear	No	No
Lirici [44]	No	Yes	No
Mavroforou et al. [67]	No	Yes	No
Narsinh et al. [33]	Yes	No	Yes
Nestor & Wilson [36]	Yes	No	Yes
Patel et al. [68]	Yes	No	No
Patel et al. [69]	Yes	No	No
Platis & Zoulias [30]	Unclear	Yes	No
Polk et al. [70]	Yes	Yes	Yes
Saniotis & Henneberg [37]	Yes	No	Yes
Satava [71]	Unclear	No	No
Schlottmann & Patti [72]	No	Yes	Yes
See et al. [73]	Yes	No	No
Senapati & Advincula [74]	No	Yes	No
Shahzad et al. [75]	Unclear	No	No
Sharkey & Sharkey [38]	No	No	Yes
Sharkey & Sharkey [76]	Yes	No	Yes
Sheetz & Dimick [43]	Yes	Yes	No
Siciliano & Tamburrini [77]	Yes	No	Yes
Siqueira-Batista et al. [78]	Yes	No	No
Smith [79]	No	Yes	No
Smyth et al. [40]	Yes	No	No
Spiers et al. [80]	Yes	No	No
Spillman & Sade [81]	Unclear	No	No
Stanberry [82]	No	No	No
Steil et al. [83]	Yes	No	Yes
Strong et al. [84]	No	No	Yes
Sullins [85]	Unclear	No	Yes
Swarnalatha & Menon [86]	Yes	No	No
Teller et al. [39]	Yes	No	Yes
Thomas et al. [87]	Yes	No	No
Tzafestas [88]	Unclear	No	No
van der Waa et al. [89]	Unclear	No	Yes
Vilanilam & Venkat [90]	No	Yes	No
Whiteside [91]	No	Yes	No
Wightman et al. [91]	Yes	No	Yes
Woo et al. [92]	No	Yes	No
Zorn et al. [93]	No	No	No

*Minor work includes comments, letters and editorials

Beyond patient benefit and harm, the assumed benefits for surgeons are also considered part of the ethical calculus [33, 35, 39, 46, 83]. This includes, for example, improvement of physician skills (e.g. suppression of hand tremor), avoidance of errors through fatigue, inattentiveness, and stress as well as shortened learning curves in some procedures. Based on a case example, Angelos [46] argues, that in some cases, empirical data supports the conclusion that using a robotic procedure may benefit only the surgeon, for example, as a way to train and maintain their skills or to gain experience with a less demanding procedure, while other manual options with equal outcomes would be available to the patient. This raises the additional question whether it would be acceptable to offer such a procedure to a patient purely for the benefit of the surgeon [46].

#### Responsibility and control

With regard to responsibility, most authors agree that being able to assign responsibility is a normative requirement, no matter the circumstances. Failing to be able to do so would otherwise result in so-called responsibility gaps [77, 89]. This term refers to a situation in which no addressee of a moral claim can be determined. Cases of responsibility gaps present serious ethical problems. Some authors, however, highlight that the detailed ethical interpretation of the responsibility principle, “raises special ethical issues in the context of increasing autonomy of medical robots where physicians are no longer in control of each and every aspect of medical procedures on the human body” [77].

The primary variable in questions of responsibility is the ability to exert any form of normatively meaningful control over the devices and, thus, is tightly connected to their level of autonomy [27, 77]. In this context, autonomy does not denote a moral good worthy of protection but is understood in a descriptive sense as a functional capacity. Nyholm defines functional autonomy as the degree to which a device is capable of creating a representation of its surroundings and to make decisions on how to reach a predefined goal [99]. With increasing degrees of functional autonomy, that is, increasing active potential, questions of responsibility become more pressing [37, 77, 83, 89].

Various typologies and categories of robotic surgical devices have been proposed by authors of our dataset to highlight significant differences in these capacities. These include categories ranging from passive to active in which robots are increasingly involved in more invasive tasks [52], different modes of control (planning and execution vs. supervised mode) [33, 37, 57] or different forms of supervision [36, 38]. This range is also mirrored in a respective conceptual language in addressing the devices, from “slave systems” or “master–slave systems” to the ascription of agent-like properties (e.g. “new players”, “mediators”) [29, 33, 47, 57, 77]. Others refer to the classification of Yang et al. noted above [77]. All these classifications aim to highlight morally relevant differences in the ability to exercise control over the devices and set the stage for in-depth inspections of environmental conditions or human–machine requirements for control, as well as specific obligations under specified circumstances.

A number of authors in this strand of the debate devote their work to exploring the topic of retrospective responsibility [27, 28, 52, 57, 77]. Retrospective responsibility is concerned with ascribing of responsibilities after the facts, that is, when harm to the patient has occurred during a procedure or a step of the procedure. Datteri argues that, in this case, someone needs to be identified to be blamed (i.e. bearing, what Datteri understands as, moral responsibility), and someone (else) should be held liable for compensating the damage [28]. The argument seems to imply a distribution or compartmentalization of different functions of responsibility. Others add that duties to compensate need to be distributed evenly and fairly between different parties involved, such as hospitals, manufacturers or human agents [27, 77]. However, it remains unclear what constitutes a fair distribution of responsibilities and whether this introduces a normative criterion to compensate for ascription problems and to avoid responsibility gaps due to a lack of control.

On the prospective side of responsibility, that is, with regard to the duties surgeons bear in preparation, implementation, and prospective decision making, there are various perspectives. According to Wightman and others, a surgeon’s prospective responsibility in participating in RAS can be primarily determined on grounds of their duty to avoid harm to the patient [29, 81]. This includes the decision to offer a robotic surgical procedure to a patient [32], setting appropriate conditions and procedures for informed consent, being aware of the specific problems connected to a robotic surgical approach, and possessing the ability and readiness to step in and take full control in case of malfunctions and errors [31].

#### Surgical learning and training

Given the complexities of surgical learning [100], credentialing and standards for introducing new procedures, this field includes arguments highlighting the responsibility of surgeons to acquire new skills and contribute to the introduction of new procedures [58, 64, 93, 101]. The arguments heavily rely on the concept of a surgical learning curve (LC) through case-based exposure and gradual increase in volume. LC is defined as the span of time (the number of cases) required for a surgeon to acquire competence in a procedure [31]. Competence is defined as the proficiency to perform a new procedure in a reasonable time and with favorable outcomes, as well as by having the expertise to react to unforeseen and unplanned events [31].

It is commonly agreed that the RAS-LC differs from other surgical procedures. Transition from manual surgical procedures to RAS, requires extensive knowledge and skills with regard to the former. However, it also typically requires experience around 5–20 cases with RAS to become proficient, while the LC in total may extend up to 100–250 cases, depending on the procedure, related experience of the surgeon, training, attitude of the surgeon, and confidence [31, 41, 54, 62, 64, 101]. Dimick and Sheetz argue that surgical credentialing programs typically require a number of proctoring cases as a proxy measure for competence, which is considerably lower than what is needed to reach proficiency. The authors report two cases to be sufficient in some RAS training programs [43]. This is in line with recent research analyzing 42 training policies from the US and reporting a mean of 3.24 cases (with a range of 1–10 cases) for initial credentialing [102]. Thus, a gap exists between acquiring credentials to use RAS and acquiring true competency and expertise [43, 64].

Secondly, irrespective of credentialing, procedures at an early stage of the learning curve are inevitably connected to a higher risk compared to later stages and may not meet the same quality standards [62, 65]. In this way, surgical learning is closely intertwined with bioethical questions of patient harm and benefit [31, 38, 58, 64, 76, 81, 91]. Weaknesses in standardized learning and credentialing procedures put responsibility on surgeons to actively seek and acquire the necessary skills and to ultimately decide when they are sufficiently prepared to conduct procedures independently [32, 79, 80].

The two above-mentioned categories of surgeons’ responsibilities and considerations regarding the learning curve intersect especially when it comes to procedures of informed consent. A key question that is heavily debated is whether it is the surgeons’ responsibility to (proactively) disclose their level of experience in procedures of informed consent. Most authors agree that surgeons should disclose their experience with RAS to patients [29, 39, 43, 45, 54, 65, 80, 85, 92]. They conclude that the question as to whether surgeons feel sufficiently trained and competent should be openly discussed alongside other important information [80]. Its discussion with patients is based on the duty to respect patient autonomy [92]. This line of argument is backed up by empirical data gathered by Lee Char et al., who found that patients perceived it as essential to know if the surgeon performs a procedure for the first time [65]. It should be noted, however, that this line of argument might raise objections of double standards. It is commonplace in medical practices that an outcome varies with experience, while extensive self-reporting of one’s experience is uncommon (and fallible). An argument would therefore be needed to justify a deviation from these norms. In addition, it remains questionable whether a duty to disclose experience should also apply to transitions from less demanding to more complex procedures (or vice versa), which is common for the surgical process of learning [43]. Finally, Sullins notes that less experience with RAS might also affect surgeons’ ability to effectively disclose all relevant risks, as they might not be fully aware of them [85] rendering it questionable whether and to what extend such information would add to the capacity of patients to decide autonomously.

#### Translational questions: introducing new procedures

The theme of translational questions is concerned with determining ethically acceptable conditions for the introduction of new devices. As noted above, the process of surgical innovation “falls into a middle range between minor modifications and surgical research” [32]. It is described to occupy a grey zone with fewer regulations as compared to other branches of medicine [58, 85, 90]. Within this grey zone, unethical experimentation—adopting new procedures with the aim of contributing to a generalizable knowledge without being able to reliably predict outcomes—must be avoided from an ethical perspective [32, 62]. Responsibility resides with the surgeon to adopt new practices to the benefit of patients while at the same time refrain from using potentially dangerous or harmful practices [38]. In addition, there is a scientific responsibility to track outcomes, ensure appropriate documentation and share this information. This obligation is often seen as a first step in more systemic efforts to ease the pathway of introducing robotic surgical devices, where manufacturers, hospitals and other institutions also carry additional responsibilities. It implies carefully timing the surgical innovations: waiting until sufficient data is available to support their use while not delaying its introduction unnecessarily and risking depriving patients of potential benefits [31, 39, 84, 90].

Although the requirement of a reasonable balance of safety, benefit, and timely adoption seems straightforward, determining all relevant factors with sufficient certainty is challenging. Besides noted methodological problems and data availability, the so-called Collingridge dilemma adds further complication [83]. In short, the Collingridge dilemma highlights the observation that shaping technology is easier in its early stages of adoption, but once established, it can become almost impossible. On the other hand, however, in the early phases uncertainty about outcomes is typically very high and, hence, hampers potential decision-making processes, while corridors to influence technologies in later stages with lesser uncertainties shrink or even become non-existent [83].

#### Financial incentives and economic pressure

As many authors note, the process of surgical innovation is not only multifaceted and rich in itself but is also put under external pressure emerging at the intersection of healthcare, and technology as business. According to Sullins it can be argued that there should be a separation of industry concerns from medical care. However, it also needs to be acknowledged that this is hard to maintain in surgical practice [85]. The fact that robotic surgical devices come with a high monetary value attached and are introduced with the necessity of generating profit translates into an ethically problematic influence on the process of surgical innovation. Some authors express concerns that in light of the high costs of devices’ maintenance and training, adopting institutions have a strong incentive to further a constant and broad use of RAS and may incentivize or even pressure their employees to comply in order to ensure adequate monetary revenue [32, 85]. Especially in market-oriented and competitive healthcare systems or in competitive disciplines of surgery, further pressure may arise out of the need to compete with other entities or colleagues [37, 38, 76]. Whether and to what extent such pressures actually exist is an empirical question that is not addressed sufficiently in our dataset. However, many authors argue that it must be part of surgeons' ethos to be aware of and to withstand these pressures [32], thereby maintaining an intact and trustworthy relationship with patients that is not unduly influenced by economic motives.

While this argument puts surgeons in the position of gatekeepers to the best of their patients as intrinsic part of their professional responsibilities, many authors critically remark financial and cooperative ties of surgeons with the industry. In addition, it has been noted that aggressive direct-to-consumer advertising campaigns [87], increased power of manufacturers through quasi-monopolization [31], and misleading or exaggerated quality claims [38] may pose ethical problems that may distort the conditions allowing for decisions in the patients’ best interest.

#### Justice in RAS

The factor of economic costs of RAS in connection with uncertain perspectives on harms and benefits, raises complex questions from a perspective of justice. The first question is, how to rate the fact that only some medical facilities may be able to afford the investment in RAS devices. Some authors point to empirical evidence showing that RAS interventions can lead to inequalities and that patients are predominantly caucasian male, located near teaching or university hospitals [31, 58]. However, as Decker notes, only if an analysis of costs and benefits comes to a positive conclusion, it may be a requirement of justice to make such an intervention accessible and affordable to all [27]. De Angelos argues more detailed that it would not be unjust to allow RAS as a procedure of elective choice, meaning that patients would have to bear additional costs, while it may constitute an injustice if one was to believe that a RAS intervention would come with additial benefits to patients [45].

Sharkey, again pointing to the high costs of RAS, on the other hand argues that a fair distribution of health care resources requires to limit expenditures to interventions with proven safety and efficacy. Costly surgical interventions like RAS may divert resources from more cost-effective options. Teller et al. expand on this by suggesting that even if a procedure proves effective and safe, it is important to consider who will have to bear the additional costs to minimize inequities of care or exclusion of patient populations [39].

Remarks like these set the stage for broader debates about distributive justice as a matter of sharing benefits and burdens within a population but also for a global justice perspective. Some authors find that especially telesurgery programs could provide the opportunity to reach distant locales and may alleviate many of the existing disparities in the delivery of surgical care [47, 59]. Hutchison et al, however, argue that RAS requires high investments and constant training of surgeons to be cost-effective, which may only be possible in densely populated areas [41]. Finally, Anvari sees RAS and its telesurgical subfields as an opportunity to connect to distant colleagues and to provide knowledge and support in a longer run [47]. Sullins, however objects by highlighting that especially countries of the global south may not be in a position to afford RAS and thus rely on more traditional approaches, making collegial support and skill transfer more difficult [85].

### Relational aspects: human–machine and patient-professional relations

Steil et al. and deTogni note that especially increased functional autonomy may not only magnify questions of responsibility but may also raise ethical questions regarding human–machine-interaction in a broader sense [52, 83]. This includes considerations around ethical team work, the human dimension in relations between surgeon and patients, and the relation between health professionals and other actors.

Beyond specific devices, ethical issues of alignment to technology and growing dependencies are noted as potential ethical concerns. Complex technical arrangements such as RAS are very likely to lead to an alignment of surgical procedures with the requirements of robotic systems, in which surgeons and team members need to acquire new skills [52, 83]. This can lead to a form of codependence—and on the negative side—may create strong incentives to rely heavily on the machines’ capabilities. An example would be a shift in the burden of proof when a surgeon wants to depart from a suggested course of action by the machine and aims to overwrite partial machine control [85]. It may also lead to a gradual decline in the manual skills of surgeons [83]. Based on the example of neurosurgical procedures, Sanniotis and Henneberg describe this as a loss of a habit of routine that is necessary for surgeons to acquire and maintain their skills. Adopting this habit, however, as well as developing and maintaining ones’ skills to ones’ best abilities is understood to be a way to comply with the do-no-harm principle [37]. As long as robots are available, this would not have negative consequences, however, if circumstances arise making manual intervention necessary, it would be detrimental to human life [37].

Taking this line of argument one step further, Sullins warns that phenomena of reverse adoption need to be closely observed. Reverse adoption describes a common tendency in the introduction of technologies in which social norms, practices and relations are changed and human agents adapt to serve the functional needs of devices, instead of devices adapting to serve users in fulfilling their goals [85]. This subordination can be understood as a degradation of autonomy, in which human users transform from those being able to set goals to a means of maintaining technical function. It is, hence, noted that especially on higher levels of functional autonomy the question of RAS presents an analytic as well as an ethical challenge to define acceptable pathways of human–machine cooperation in the operating theater that preserve the human capacity to determine the ends. According to Sullins, the first question is to define what it means to be autonomous in this context while second is to clarify how this autonomy is compatible with increasingly autonomous machines. The answer, as is argued, lies in understanding cooperation as an ethical question that becomes increasingly pressing with more complex devices. It should be explored by outlining desirable forms of cooperation. Depicting forms of human–machine cooperation as a gradual continuum, positive notions of cooperation remain rather unclear and seldomly extend beyond ideas that connect to the theme of meaningful control. On the negative side, instrumentalization described as a degradation of human actors from those who set ends to those who serve as means to ensure functioning of the machines [27] is understood to be unethical and a form of cooperation to be avoided.

With regard to the professional-patient-relationship, authors acknowledge the importance of human-to-human contact as part of the surgical process [28, 33, 35, 36, 38, 76, 83]. The theme most discussed is whether distance between surgeons and patients may affect mutual commitments [85], potentially weakening or disrupting trust and relationship between patients and professionals (43,62). Objectification of patients, i.e., the reduction of patients to objects of surgery rather than active participants, should be avoided [36, 85], especially in telesurgery. However, taking potential benefits into account as well as the fact that potential phenomena of distancing may not extend beyond the operating procedure itself, the authors in our dataset seem to be less concerned about the impact on the professional-patient-relationship.

## Discussion

The main themes presented in our results testify to a broad range of different ethical considerations and perspectives on RAS. In what follows, we comment on these findings in relation to our aims of developing a systematic overview of the ethical issues and to trace connections to technology and technological properties within these debates.

Regarding our first aim of systematizing the debate, avoidance of harm, advancing patients’ interests, and maintaining control and responsibility over the process of RAS seem to be commonly accepted primary ethical reference points. Most authors in our dataset confine themselves to addressing only one of these issues. Therefore, it remains unclear how these different principles relate to or complement each other, or how they might be balanced in case of conflict. Based on the generalized themes of this review, we suggest that understanding each of these themes within the context of a professional surgical ethos could help systematize the debates and provide further insights.

McCullough et al. have argued that the surgical ethos sets particularly high standards, drawing on the distinct circumstances of surgical interventions [103], including the proximity of surgeons and patients, the necessity to harm before healing, and the patients’ acceptance of and surrender to these conditions. The authority of surgeons over this process is understood as a necessary requirement. However, from an ethical perspective it can only be granted conditionally and is only justified if surgeons uphold the position of a trustworthy fiduciary of patients’ interests while displaying and maintaining the requisite skills, knowledge, and practical judgement as virtues of the surgical profession [103].

Against this background, our review indicates that RAS introduces changing conditions for maintaining legitimate authority in the operating theater. For instance, unclear patient benefit or questionable trustworthiness of evidence in regard to patient benefit do not only point to potential research gaps, they also threaten the position of surgeons as moral fiduciaries, necessitating ethical considerations. Likewise, questions of learning and questions of control point to surgeons techné and knowledge as requirements controlling and using RAS devices properly. In addition, this view shows that these standards might be riddled with additional complexities and conflicting requirements that need to be mitigated through proper judgement. For instance, while timely adoption of RAS and proficiency through increasing case volume are encouraged, there is also the call to uphold traditional skills through constant practice, and to maintain the competence to step in if errors occur. Mandating both as part of the robot surgeons' ethos is demanding. It requires further deliberationto reconcile both duties to avoid overburdening the surgical role with conflicting obligations.

These considerations show that although RAS might rely on commonly accepted ethical principles, satisfying the high standards of surgical ethos requires adapting these principles to the circumstances of RAS. This includes re-evaluating and re-orienting surgical duties and professional ethos in light of new and emerging technological possibilities, as well as identifying and mitigating value conflicts that arise with these shifts. Looking to the future, we agree with authors in our dataset who suggest a gradual shift that prescribes developing and maintaining technical knowledge, competences and literacy in data and digitization as part of the virtues for robotic surgeons, in addition to their traditional skillset to meet these challenges.

With regard to our second aim, that is, to understand how RAS and its technical properties are addressed and linked to ethical dimensions in the literature, we observe two differing perspectives. This becomes apparent when comparing the analytic lenses through which devices are examined in questions of responsibility and relational effects. As noted above, a significant part of the included papers refers to RAS in a conceptual language of “master–slave” devices, “slave systems” or (enhanced) tools. These tools are described as means to a specific end, with a low degree of autonomy, entirely dependent on the human operator. With this passive role of the system, a clear distinction between humans and machines is implied, since robotic surgical devices are understood to merely translate hand movements into movements of mechanical parts while fully respecting the surgeon’s intentions, goals, and strategies. This perspective is equated with a “division of labour” in which the human operator is in control by setting and defining the rules, while the machine is confined to executing these. Consequently, the devices are understood as an extension of surgeons’ physical and volitional capacities, which are completely at their disposal [89]. In the philosophy of technology, this view or its derivative perspectives are often described as an instrumental perspective in which technologies appear as means to an end within human activity.

The second perspective, however, highlights the interaction, interplay, and cooperation between human actors (e.g, the human surgeons or patients) and the robotic devices. This view situates the machines as physical entities with information processing capacities in the operating theater [52]. From this perspective, relationality is seen as an important feature [52, 83]. For example, while the Da Vinci robot translates human movement into the movement of robotic instruments, it also provides surgeons with haptic feedback, thus taking the position of a physical mediator between human surgeons and patients. The robotic movements are intertwined with the surgeons’ as well as the patients’ bodies, integrating with the surgeon-patient-interaction. This mediating position is understood as reshaping this interaction in a novel way [52]. With this shift, a distinct active potential of robotic surgical devices in their role as mediators based on their information processing capacities is noted. The devices do not only transmit but actively transform what is and can be done from the surgeons’ perspective. This view, hence, distances itself from understanding RAS devices as passive machines [77]. Examples of this active potential include tremor suppression of surgeons’ hand movements or restricted movement path in newer systems [38], as well as preprogrammed trajectories [37], that execute or modify human actions [57]. As, for example, Di Paolo concludes, RAS integrates a “new player” in the operating theater [54]. This new player breaks down the clear and embodied boundaries and sensations between patients and surgeons through its bidirectional interaction [52], making division of labor and control less clear [83], and creating distinct and sometimes disruptive changes in human practices.

Proponents of the instrumental perspective are confident that using RAS does not differ substantially from traditional interventions without robots [78]. With regard to responsibility and professional ethos they argue that only very few new questions besides potential harms and benefits arise, as compared to laparoscopic or open procedures [78, 79]. The operating surgeon is seen as being fully in control and responsible [36, 44, 78, 79]. Consequently, the ethical perspective that emerges from an instrumental view is mostly based on considering trade-offs between improvement of patient care and potential losses due to the means used in specific conditions.

However, while this perspective seems to be intuitively plausible, it does not account for the effects and implications of using RAS that are more difficult to quantify in terms of health outcomes or that arise as a consequence of changing interactional patterns. In addition, it does not seem to be fruitful to consider the effects of devices gaining a more active role as described above. While an instrumental perspective may be suitable for devices that have low levels of functional autonomy, it becomes inadequate as device activity increases. By contrast, authors following the second perspective shift their focus on the mediating position robotic devices occupy in the operating theater. This view is, hence, tightly connected to questions of control and professional obligations of surgeons, leading to considerations around human–machine-interaction, inter-team relations, and relations between patients and medical professionals.

Based on these considerations, we understand the way devices are conceptualized as an important precursor of ethical analysis, as it provides a focal lense through which RAS is addressed ethically. However, we suggest that framing this as a mere ontological question that needs to be settled to provide adequate ethical analysis would be an oversimplification. Instead, such questions should be addressed at a conceptual level. It can, hence, be understood as a question of so-called conceptual ethics or conceptual engineering in approaching RAS [104]. Conceptual ethics, broadly construed, is concerned with reflecting on the choice of representational devices whenever that choice may have non-conceptual consequences. The decision to use—or not use—a particular concept can be based on a set of values appropriate to the context and goals of use. With this in mind, we suggest understanding the different perspectives in our dataset as offering complementary focal lenses to the phenomena of RAS, which sometimes overlap without being mutually exclusive and may be more or less suitable depending on the context and goal in which they are put to use.

With regard to a future perspective on the analysis of RAS that aims to anticipate upcoming ethical issues, questions of conceptual ethics warrant further inspection and careful reflection. The current technological development has allowed for a—mostly implicit—instrumental perspective to satisfy the conceptual conditions for a fruitful and comprehensive analysis. While these conditions may be easier to satisfy with devices with no or low functional autonomy, the likely shift towards more active and autonomous devices calls for reflective caution with regard to the presuppositions of future ethical inquiries to avoid missing important questions. For instance, questions of control and responsibility require to loosen the tight grip of thinking in means-ends-schemes. Providing answers in these matters requires rethinking conditions of control and, hence, to consider the broader picture of interactional patterns that allow to transfer control back and forth, or to step in and take over control (for example in terms of feedback loops, overriding mechanisms, timing windows for decision making, etc.) [77]. This points to surgeons’ responsibilities in contributing to developmental steps to create such conditions [101].

### Strength and limitations

Our paper provides a thorough and systematic review of the ethical issues surrounding RAS, covering a wide range of literature from various academic fields. With this, we integrate perspectives from philosophy, medical ethics, philosophy of technology, medicine and computer science to offer a comprehensive overview. We must concede, however, that our findings come with at least two limitations. First, caution is warranted with the criterion of operationalizing different levels of functional autonomy of devices which proves more difficult than expected and may have had an impact on the inclusion and exclusion of studies. Secondly, it is likely that our search strategy and selection of sources may have resulted in a cultural bias in our dataset, limiting the generalizability of the findings. Although we did not exclude studies based on language, our dataset contained very few non-English and non-Western records, making it more difficult to identify ethical issues based on culturally different perspectives of RAS or medicine and care in a more general sense. With this in mind, we do not understand our results to be exhaustive at this point.

## Data Availability

The data used in this review is available upon reasonable request from the corresponding author.
